# Non-pharmaceutical interventions and epigenetic aging in adults: Protocol for a scoping review

**DOI:** 10.1371/journal.pone.0301763

**Published:** 2024-08-19

**Authors:** Alina Liebich, Shenglin Zheng, Theresa Schachner, Jacqueline Mair, Mia Jovanova, Falk Müller-Riemenschneider, Tobias Kowatsch

**Affiliations:** 1 School of Medicine, University of St. Gallen, St.Gallen, Switzerland; 2 Saw Swee Hock School of Public Health, National University of Singapore and National University Health System, Singapore, Singapore; 3 Future Health Technologies, Singapore–ETH Centre, Campus for Research Excellence And Technological Enterprise (CREATE), Singapore, Singapore; 4 Department of Management, Technology, and Economics, ETH Zurich, Zurich, Switzerland; 5 Digital Health Center, Berlin Institute of Health, Charité-Universitätsmedizin Berlin, Berlin, Germany; 6 Institute for Implementation Science in Health Care, University of Zurich, Zurich, Switzerland; UNAM Facultad de Estudios Superiores Zaragoza: Universidad Nacional Autonoma de Mexico Facultad de Estudios Superiores Zaragoza, MEXICO

## Abstract

**Introduction:**

Aging is the strongest risk factor for most chronic diseases. The rising burden of an aging population and non-communicable diseases (NCDs), contributes to escalating costs for society. Several non-pharmaceutical interventions can lower the risk of NCDs, including common mental disorders (CMDs), and may slow down biological aging, as evidenced by outcome markers such as epigenetic clocks. However, a comprehensive overview of whether and which non-pharmaceutical interventions may impact human epigenetic aging is missing. Synthesizing evidence of interventions on epigenetic aging that can be adopted by a wider population is key to guide healthy aging initiatives and to reduce the burden of NCDs and CMDs. This scoping review will identify and assess non-pharmaceutical interventions aimed to slow down epigenetic aging, including their intervention components, and the mode used for intervention delivery.

**Methods and analysis:**

This protocol will include single- and multicomponent intervention studies that target individuals ≥ 18 years of age and use epigenetic clocks as primary or secondary outcomes. Five electronic databases will be searched for studies between July 2011 until December 2023. The final search will include the search terms adults, non-pharmaceutical interventions, epigenetic aging and their respective synonyms. We will include randomized controlled trials, non-randomized controlled studies, cohort studies, and case-control studies. Additionally, the reference list of other reviews will be screened for relevant articles. Study selection is carried out based on the defined eligibility criteria by two authors. Quality and risk of bias for the included studies will be assessed using the Critical Appraisal Skills Programme (CASP) checklist. Data extraction will include generic key information such as the research question and results, the intervention components, and specific epigenetic outcome measures used. Further data regarding the delivery mode of the treatment protocol will be collected.

**Ethics and dissemination:**

This scoping review will summarize the characteristics of non-pharmaceutical intervention studies on epigenetic aging. This review will be the first step to formally identify key intervention components and delivery modes to guide future research on healthy aging interventions. The results will be disseminated through a peer-reviewed publication and presented at relevant conferences. This review will synthesize information from available publications and does not require further ethical approval.

**Registration details:**

Open Science Framework https://doi.org/10.17605/OSF.IO/FEHNB.

## Introduction

Aging is the strongest risk factor for most chronic diseases [1]. Even though life expectancy has increased globally between 2000 and 2019 by approximately 6.6 years, the number of years lived in good physical and mental health has failed to keep pace [2, 3]. Recent trends point to an expansion of morbidity [4]. This discrepancy between life expectancy and the period of life in good health can largely be attributed to the rising prevalence of non-communicable diseases (NCDs) and common mental disorders (CMDs), such as cardiovascular disease, diabetes, and cancer predominantly caused by aging of the population on one side, and the global spread of the well-known Western lifestyle, on another side [5–7]. The rising burden of NCDs, and CMDs, as well as the aging population globally, constitute primary drivers for increased healthcare expenditures and long-term care costs, positing significant challenges to healthcare systems and economies worldwide [8–10]. Healthy aging describes a status when years of good health approach the biological lifespan, leading to a compression of years lived with disability and gaining healthy years [11]. Several factors, including good physical, mental, and cognitive health, having few medical conditions, and social well-being, have been identified as important determinants of healthy aging [12, 13]. Thus, understanding which interventions, readily adoptable by a wider population, promote healthy aging offers a higher quality of life for the individual and benefits the whole society [14].

Aging is associated with the cumulative breakdown of multiple physiological systems, which makes the organism more vulnerable to disease later in life [15]. While chronological age refers to the years a person has been alive, biological aging can be defined as the progressive, event-dependent decline in the ability to maintain biochemical and physiological functions [16]. Multiple molecular mechanisms, such as chronic inflammation, DNA damage, dysfunctional mitochondria, or increased senescent cell load, constitute the pathophysiology of many age-related conditions [5, 17]. These aging-related pathways can be used to predict biological age and offer a target for intervention [18–20]. The geroscience hypothesis proposes that targeting the aging process rather than the individual diseases of aging can delay or even prevent the onset of many age-related diseases [1, 21]. Currently, strategies to alter the biological aging process are being developed, whereby anti-aging drugs are mostly limited to preclinical lab trials [22]. There has been increasing evidence that non-pharmaceutical factors can slow down, or even reverse, biological aging and extend the time lived in good health [23]. Non-pharmaceutical interventions can be defined as any intervention intended to improve the health or the well-being of individuals that does not involve the use of drugs or medicine and is either composed of a single component or a multicomponent intervention [24]. More specifically, several lifestyle factors, such as regular physical activity, abstinence from smoking, maintaining a nutrient-rich diet, and low alcohol consumption, have been associated with substantial gains in years free from chronic diseases and disabilities [25]. Additionally, maintaining a strong sense of life purpose [26], strengthening social connections [27], brain stimulations [28], preserving the circadian rhythm [29], exposure to cold temperatures [30, 31] and calorie restriction [32] are identified as promising components to reduce the risk of morbidity in old age. Fitzgerald et al. developed a multicomponent treatment program that included a dietary, sleep, exercise, and relaxation intervention. They measured a 3.23-year decreased biological age in the intervention group compared to the control group using Horvath’s online DNAmAge clock [23]. Non-pharmaceutical interventions to promote healthy aging are beneficial as they are non-invasive, accessible, have minimal or no adverse effects, and are mostly low-cost [22, 33].

Various measurement instruments such as questionnaires, performance tests, and biomarkers have been used to measure the effectiveness of healthy aging interventions [34]. Biomarkers of biological age provide a more comprehensive picture of one’s overall health compared to an individual’s chronological age [35–37]. Further, biological age predictors have the potential to reflect the impact of an intervention on the individual aging process before sufficient clinical events have accumulated, making them a valuable measure of the effectiveness of healthy aging interventions [38, 39]. Among all biological age predictors, epigenetic clocks are considered one of the most promising [19]. Epigenetic aging refers to the measurement of modifications to the DNA that can affect gene expression without altering the DNA sequence itself [40, 41]. It is typically measured using epigenetic clocks, which analyze specific patterns of DNA methylation [42]. These clocks provide a numerical estimate of an individual’s biological age, reflecting the cumulative effects of aging-related changes in gene regulation [43, 44]. Since 2011, when the first epigenetic clock by Bocklandt was introduced, multiple other epigenetic clocks based on different biological samples and statistical modeling methods have been discovered [45, 46]. They have shown strong predictive power in assessing morbidity and mortality risk, making them the most reliable predictor for aging-related processes [19, 38].

To date, no comprehensive synthesis of the evidence has explored non-pharmaceutical interventions for modifying epigenetic aging in human adults [23, 47–49]. Previous reviews have focused on pharmaceutical interventions and often included animal studies [37, 49, 50]. We aim to map and synthesize the evidence of non-pharmaceutical interventions on epigenetic aging in human adults, including the key intervention components, delivery modes, and outcome metrics for epigenetic aging. Compared to previous works, we hereby aim to identify readily implementable strategies that benefit the targeted human adult population so that limited resources can be used effectively in future study designs and eventually guide scalable public health initiatives in implementing evidence-based non-pharmaceutical interventions [49, 51]. To this end, we ask the following research questions:

1. Which key components have been included in non-pharmaceutical interventions targeting the deceleration of epigenetic aging in humans?
2. What delivery modes have been used to administer these interventions?
3. What outcome measures have been used to assess the effectiveness of intervention on epigenetic aging?

## Methods and analysis

This protocol is based on the Preferred Reporting Items for Scoping Reviews (PRISMA-ScR) [52] and will follow the JBI (Joanna Briggs Institute) manual for evidence synthesis [53]. The protocol was written before the review activities and registered in Open Science Framework (OSF) (https://doi.org/10.17605/OSF.IO/FEHNB).

### Eligibility criteria

The eligibility criteria are summarized in Table 1. They were defined using the PICOS framework including population, intervention, control, outcome and study type (PICOS) [54].

**Table 1:** A list of all inclusion and exclusion criteria using the PICOS framework.

**Inclusion criteria**		
**Population**	Adults	18 years of age or older
**Intervention**	Non-pharmaceutical interventions	Any intervention intended to improve the health or the well-being of individuals that do not involve the use of any drugs or medical treatments
**Control**	Not specified	No intervention
		Any control groups such as usual care, waitlists, and other interventions in any format
**Outcome**	Epigenetic aging	Any measurement of epigenetic alterations that is intended to predict epigenetic age
**Study Type**	Study design	Randomized controlled trials, non-randomized controlled trials, cohort studies, and case-control studies
**Other**	Language	English
	Publication Date	From 2011 until December 2023
**Exclusion criteria**		
**Population**	Children and adolescents less than 18 years of age; pregnant women	
	Animals	
	Indirectly affected groups of people (E.g. relatives, medical professionals, or parents)	
**Intervention**	Interventions that included components that are only available on prescription or are considered drugs	
	Interventions where non-pharmaceutical intervention was not the primary intervention component	
**Control**	Not specified	
**Outcome**	Non-pharmaceutical interventions that reported other biological age predictors	
**Study Type**	Non-peer reviewed papers, conference papers, letters to editors, dissertations, qualitative studies, study protocols, reviews, case reports, case series, expert opinions or reports of expert committee	
	Full-text not accessible	

#### Population

We will include studies targeting adult populations aged 18 years of age or older, including healthy individuals and clinical patients. Studies involving animal subjects, as well as those targeted at children, adolescents, or pregnant women will be excluded.

#### Intervention

The target intervention of this scoping review includes non-pharmaceutical interventions. A non-pharmaceutical intervention will be defined as any intervention intended to improve the health or the well-being of individuals that does not involve the use of any drugs or medicine [55, 56]. Included interventions are single- and multicomponent interventions. Example components are the following: (1) physical activity (e.g. reaching a specific step goal), (2) diet (e.g. fiber-rich food intake), (3) dietary supplements [57] (including nutrition supplements and nutraceuticals [58]), (4) interventions aimed to reduce substance use (including smoking and alcohol consumption), (5) mental health exercises (e.g., meditation or slow-paced breathing exercises), (6) cognitive health exercises (e.g., memory exercises), (7) interventions aimed to promote social integration (e.g. peer education and advocacy through recreation and leadership [59]), (8) sleep hygiene interventions (e.g., sleep education [60]) and (9) other non-pharmaceutical interventions (e.g., exposure to cold temperature). The search will not be limited to a specific intervention setting or region, the time frame, or the follow-up period of the intervention.

#### Control

This review does not specify a comparison or control group.

#### Outcome

The outcome is epigenetic aging measured by epigenetic clocks. Epigenetic clocks are based on computational models that use an individual’s DNA methylation pattern measured for example from a blood sample and may predict a person’s biological age [46]. Thus far, epigenetic clocks are considered the most promising biological age predictors and have outperformed other biological age estimators regarding predictive power in assessing morbidity and mortality risk [19]. DNA carries the genetic material responsible for building an organism and is regulated by epigenetic activities that refer to chemical modifications such as methylation of DNA sites and hereby controlling gene expression [40, 41]. Literature has identified age-related methylation patterns at particular regions of DNA called CpG sites [42]. Cytosine-5 methylation levels at CpG sites are the base for multivariate machine learning models that predict epigenetic age [43]. We will consider all verified epigenetic age predictors defined as a measurement of epigenetic alterations, such as Horvath’s DNAmAge clock [42], Levine’s DNAmPhenoAge [61], Lu’s DNAmGrimAge [62] etc.

#### Study type

We will include randomized controlled trials, non-randomized controlled trials, cohort studies, and case-control studies.

### Information sources

The search will be conducted using the following databases: Medline, Embase, Scopus, APA PsycInfo and Web of Science for articles published between 2011 and December 2023, as the first epigenetic clock was described by Bocklandt in 2011 [45]. Only peer-reviewed studies published in English will be included as English is considered the leading language of medical research [63]. Additionally, the reference list of other reviews will be screened for relevant articles.

### Search strategy

The scoping review’s search strategy is developed according to the Peer Review of Electronic Search Strategies (PRESS) [64] and PRISMA-ScR guidelines [52]. An initial limited search of two electronic databases (Medline and Embase) will be conducted using the keywords “non-pharmaceutical intervention” and “epigenetic aging” to identify main descriptors, synonyms, and keywords included in titles and abstracts of relevant publications [49, 53, 65]. The generated keywords and associated mesh terms were used to calibrate the final search strategy for Medline via Pubmed using an altered PICO framework and are presented in Table 2 [66]. The final search strategy will be adapted to each database. The full search strategy is available in Table 2.

**Table 2:** Final search strategy for Medline via Pubmed using the PIO framework.

ID	PIO	Search components	Search terms
**1**	**Population**	Human adults	Adult* OR Female* OR Human* OR Inpatient* OR Male* OR Man OR Men OR Outpatient* OR Patient* OR People OR Person OR Population OR Woman OR Women
**2**	**Intervention**	Non-pharmaceutical Interventions	"Active health*" OR "Aging intervention*" OR "Aging-intervention*" OR "Ageing intervention*" OR "Behavioural change*" OR "Behavioral change*" OR "Behavioural intervention*" OR "Behavioral intervention*" OR "Health intervention*" OR “Health promotion” OR “Healthy aging” OR "Lifestyle*" OR "Longevity” OR "Multifactor intervention*" OR "Multicomponent intervention*" OR "Multi-component intervention*" OR “Non-drug intervention*” OR "Non-pharmaceutical intervention*" OR “Non pharmaceutical intervention” OR "Non-pharmaceutical strateg*" OR "Non pharmaceutical strateg*" OR “Non-pharmacological intervention*” OR “Non pharmacological intervention*” OR "Rejuvenation intervention*" OR "Rejuvenation strateg*"
**3**	**Outcome**	Epigenetic aging	“Age acceleration*” OR "Age biomarker*" OR "Ageing biomarker*" OR "Aging biomarker*" OR "Age clock*" OR "Ageing clock*" OR "Aging clock*" OR "Age estimat*" OR "Ageing estimat*" OR "Aging estimat*" OR "Age predict*" OR "Ageing predict*" OR "Aging predict*" OR "Age revers*" OR "Biological ag*" OR "Biological clock*" OR "DNAmAge" OR "DNA methylation*" OR "DNAm based biomarker*" OR "Epigenetic ag*" OR "Epigenetic mutation load"
**4**	**Other**	Research conducted since July 2011	("2011/07/01"[Date—Publication]: "2023/12/31"[Date—Publication])

The search terms will be applied as followed: ID1 AND ID2 AND ID3 AND ID4 in all fields.

### Selection of studies

Study selection is carried out based on the previously defined eligibility criteria by two authors. All bibliographic records will be imported to Citavi 6.17 to identify duplicate entries. After removing the duplicates, the first author will screen records for inclusion based on their titles and abstracts using Rayyan (https://www.rayyan.ai/). A second author will independently assess the included studies to confirm their adherence to the established eligibility criteria. A third researcher will join to resolve the discrepancies and achieve consensus. A list of all included and excluded studies and reasons for exclusion will be reported.

### Data extraction

The first author will carry out full-text inspection of all selected sources and conduct data extraction. The second author will confirm the data in a separate step to reduce the chance of errors. Discrepancies will be reported to the first author and discussed jointly. We will extract the following information: author’s name, year of publication, country of origin, study type, publication title, population type, research questions and results, intervention components, the specific epigenetic outcome measures used, data regarding the delivery mode and duration of the treatment protocol.

### Analysis of the evidence

The quality and risk of bias assessment for the included studies will be performed using the Critical Appraisal Skills Programme (CASP) checklist [67]. The quantitative evidence, including the number of studies, number of published studies, mean duration of study intervention, population characteristics, and percentage of specific epigenetic clocks, will be analyzed using descriptive statistics in Covidence and Excel. Furthermore, the average occurrence of specific intervention components and their respective subcategories (e.g., composition of diet, type of physical activity, etc.) and the specific treatment protocols to deliver the intervention will be analyzed. Qualitative data will be analyzed in a narrative format.

### Presentation of the results

The evidence synthesis will be presented through tables and diagrams. A discussion with the relevant literature and a summary will follow the summarized data that is in alignment with the objectives of the scoping review. Additionally, a map will be created with countries in which the intervention studies have been conducted.

### Ethics and dissemination

This review will synthesize information from available publications and does not require further ethical approval. The results will be disseminated through a peer-reviewed publication in an academic journal and presented at relevant conferences. It will also provide key information to other researchers, public policy makers and healthcare professionals interested in developing interventions to promote healthy aging.

## Conclusion

Our scoping review aims to synthesize current evidence on non-pharmaceutical interventions to slow down epigenetic aging, focusing on identifying intervention components and the mode used for intervention delivery. These findings could provide important guidance for the development of future interventions to promote healthy aging and carry key potential implications to alleviate the burden of NCDs and CMDs. The screening process is planned to start in January 2024 and is expected to be finalized by February 2024.

## Supporting information

S1 Checklist PRISMA-P 2015 checklist. (PDF)
