# Supplementary material for: Non-pharmaceutical interventions and epigenetic aging in adults: Protocol for a scoping review
Source: PLoS One. 2024 Aug 19;19(8):e0301763. doi: 10.1371/journal.pone.0301763 (PMC11332928; doi:10.1371/journal.pone.0301763)
Supplement: Peer review history

# Peer review: Decision Letter 0

**DOI:** 10.1371/journal.pone.0301763.r001

**Authors:** Victor Manuel Mendoza-Nuñez

22 Mar 2024

Non-pharmaceutical interventions and epigenetic aging in adults: protocol for a scoping review

PONE-D-23-44102

Dear Dr.Tobias Kowatsch,

We’re pleased to inform you that your manuscript has been judged scientifically suitable for publication and will be formally accepted for publication once it meets all outstanding technical requirements.

Within one week, you’ll receive an e-mail detailing the required amendments. When these have been addressed, you’ll receive a formal acceptance letter and your manuscript will be scheduled for publication.

An invoice will be generated when your article is formally accepted. Please note, if your institution has a publishing partnership with PLOS and your article meets the relevant criteria, all or part of your publication costs will be covered. Please make sure your user information is up-to-date by logging into Editorial Manager at Editorial Manager® and clicking the ‘Update My Information' link at the top of the page. If you have any questions relating to publication charges, please contact our Author Billing department directly at authorbilling@plos.org.

If your institution or institutions have a press office, please notify them about your upcoming paper to help maximize its impact. If they’ll be preparing press materials, please inform our press team as soon as possible -- no later than 48 hours after receiving the formal acceptance. Your manuscript will remain under strict press embargo until 2 pm Eastern Time on the date of publication. For more information, please contact onepress@plos.org.

Kind regards,

Victor Manuel Mendoza-Nuñez, PhD

Academic Editor

PLOS ONE

Journal requirements:

When submitting your revision, we need you to address these additional requirements.

1. Thank you for stating the following in the Competing Interests section:

“I have read the journal's policy and the authors of this manuscript have the following competing interests: AL, JM, MJ, and TK are affiliated with the Centre for Digital Health Interventions, a joint initiative of the Institute for Implementation Science in Health Care, University of Zurich, the Department of Management, Technology, and Economics at ETH Zurich, and the Institute of Technology Man-agement and School of Medicine at the University of St.Gallen. The Centre for Digital Health In-terventions is funded in part by CSS, a Swiss health insurer, MavieNext, an Austrian healthcare provider, and MTIP, a Swiss digital health investor. TK is also a co-founder of Pathmate Technol-ogies, a university spin-off company that creates and delivers digital clinical pathways. However, CSS, MavieNext, MTIP, or Pathmate Technologies were not involved in this protocol. All other authors declare no conflict of interest.”

Please confirm that this does not alter your adherence to all PLOS ONE policies on sharing data and materials, by including the following statement: "This does not alter our adherence to  PLOS ONE policies on sharing data and materials.” (as detailed online in our guide for authors http://journals.plos.org/plosone/s/competing-interests).  If there are restrictions on sharing of data and/or materials, please state these. Please note that we cannot proceed with consideration of your article until this information has been declared.

Please include your updated Competing Interests statement in your cover letter; we will change the online submission form on your behalf.

# Editor report: Acceptance letter

**DOI:** 10.1371/journal.pone.0301763.r002

**Authors:** Victor Manuel Mendoza-Nuñez

5 Jul 2024

PONE-D-23-44102 

PLOS ONE

Dear Dr. Kowatsch, 

I'm pleased to inform you that your manuscript has been deemed suitable for publication in PLOS ONE. Congratulations! Your manuscript is now being handed over to our production team.

At this stage, our production department will prepare your paper for publication. This includes ensuring the following:

* All references, tables, and figures are properly cited

* All relevant supporting information is included in the manuscript submission,

* There are no issues that prevent the paper from being properly typeset

If revisions are needed, the production department will contact you directly to resolve them. If no revisions are needed, you will receive an email when the publication date has been set. At this time, we do not offer pre-publication proofs to authors during production of the accepted work. Please keep in mind that we are working through a large volume of accepted articles, so please give us a few weeks to review your paper and let you know the next and final steps. 

Lastly, if your institution or institutions have a press office, please let them know about your upcoming paper now to help maximize its impact. If they'll be preparing press materials, please inform our press team within the next 48 hours. Your manuscript will remain under strict press embargo until 2 pm Eastern Time on the date of publication. For more information, please contact onepress@plos.org.

If we can help with anything else, please email us at customercare@plos.org.

Thank you for submitting your work to PLOS ONE and supporting open access. 

Kind regards, 

PLOS ONE Editorial Office Staff

on behalf of

Dr. Victor Manuel Mendoza-Nuñez 

Academic Editor

PLOS ONE
